# Methods to estimate effective population size using pedigree data: Examples in dog, sheep, cattle and horse

**DOI:** 10.1186/1297-9686-45-1

**Published:** 2013-01-02

**Authors:** Grégoire Leroy, Tristan Mary-Huard, Etienne Verrier, Sophie Danvy, Eleonore Charvolin, Coralie Danchin-Burge

**Affiliations:** 1 AgroParisTech, UMR1313 Génétique Animale et Biologie Intégrative, 16 rue Claude Bernard, F-75321, Paris 05, France; 2 INRA, UMR1313 Génétique Animale et Biologie Intégrative, Domaine de Vilvert, F-78352, Jouy-en-Josas, France; 3 AgroParisTech, UMR518 Mathématiques et Informatique Appliquées, 16 rue Claude Bernard, F-75321, Paris 05, France; 4 IFCE, F-61310, Le Pin au Haras, France; 5 Institut de l’Elevage, 149 rue de Bercy, F-75595, Paris 12, France

## Abstract

**Background:**

Effective population sizes of 140 populations (including 60 dog breeds, 40 sheep breeds, 20 cattle breeds and 20 horse breeds) were computed using pedigree information and six different computation methods. Simple demographical information (number of breeding males and females), variance of progeny size, or evolution of identity by descent probabilities based on coancestry or inbreeding were used as well as identity by descent rate between two successive generations or individual identity by descent rate.

**Results:**

Depending on breed and method, effective population sizes ranged from 15 to 133 056, computation method and interaction between computation method and species showing a significant effect on effective population size (*P* < 0.0001). On average, methods based on number of breeding males and females and variance of progeny size produced larger values (4425 and 356, respectively), than those based on identity by descent probabilities (average values between 93 and 203). Since breeding practices and genetic substructure within dog breeds increased inbreeding, methods taking into account the evolution of inbreeding produced lower effective population sizes than those taking into account evolution of coancestry. The correlation level between the simplest method (number of breeding males and females, requiring no genealogical information) and the most sophisticated one ranged from 0.44 to 0.60 according to species.

**Conclusions:**

When choosing a method to compute effective population size, particular attention should be paid to the species and the specific genetic structure of the population studied.

## Background

In population genetics, different tools are used to assess genetic diversity for conservation purposes and one of the most commonly used indicators is the effective population size (*N*_*e*_) developed by Wright [1]. *N*_*e*_ is defined as the number of reproducing individuals, bred in an idealized population in which all individuals are of the same sex and selfing is permitted, and that leads to the same decrease of genetic diversity than the population being studied [2]. However, several genetic diversity indicators have been proposed and the most classical ones are genetic drift through temporal changes in allele frequencies (variance of effective population size), increase in homozygosity (inbreeding effective population size), or the rate at which unique alleles are lost (eigenvalue effective population size) [3,4]. Moreover, different information sources (demographic information, pedigree or molecular data) can be used to estimate *N*_*e*_. Therefore, when estimating *N*_*e*_, it is important to know precisely which process is ongoing and to have the information used to assess it [3].

Until the recent development of dense single nucleotide polymorphism (SNP) chips, it was generally recommended to assess genetic variability within a population from pedigree data if available, which is often the case in captive or domestic animal populations [3,4]. On the basis of demographic/pedigree data, several methods have been proposed to compute *N*_*e*_. Ideally, they should lead to the same *N*_*e*_ estimate [5,6] but they differ in terms of which Wright-Fisher properties (among others) are considered [3]. For instance, variance of progeny size [6], an indicator of both change in allele frequency and inbreeding (at least for unselected populations), is frequently used to compute *N*_*e*_. A large number of methods also focus on the increase in homozygosity over generations by measuring Identity By Descent (IBD) probability. IBD probability represents the probability that two randomly chosen alleles of an individual are inherited from the same ancestor. Inbreeding *F* and coancestry *C* (also called kinship) coefficients are two classical genealogical estimators of IBD probability [7] that differ according to whether the considered alleles are from a single individual, or two individuals, respectively. The relation between IBD and *N*_*e*_ is based on the classical formula

$$N_{e}=1/2\text{Δ}\text{IBD},$$

where Δ*IBD* is the rate of IBD, classically estimated by the rate of inbreeding Δ*F* as Δ*IBD*, i.e. the evolution of the average coefficient of inbreeding *F* over time [2]. However, recently, new methods have been proposed to compute Δ*F* from an approximate rooting of individual inbreeding coefficients based on pedigree knowledge (Equivalent complete generations, *EqG*) [8]. Cervantes et al. [9] have also suggested using coancestry instead of inbreeding for IBD estimation.

All these methods do not differ only in terms of the indicator or force observed, but also in terms of the time scale investigated and the amount of available information. Moreover, they are more or less sensitive to the level of pedigree knowledge and to some parameters related to breeding conditions, such as the existence of population subdivisions or departure of the random mating hypothesis, which may lead to biased *N*_*e*_ estimates. Depending on the context and the authors, one or several of these methods have been applied to domestic breeds [10-14] and captive animal populations [15,16]. More specifically, the fact that in a number of breeds, no pedigree information is available, the simplest approximation of *N*_*e*_ (computed on the basis of number of breeding males and females) has been used to classify the endangerment level of breeds by the European Association for Animal Production (EAAP), and the Food and Agriculture Organisation (FAO).

Our study aimed at comparing several methods used to estimate *N*_*e*_ from pedigree data for a wide range of domestic animal populations. One hundred and forty breeds from four different species, i.e. dog, sheep, cattle and horse, were used. These include intensively selected breeds with large current population sizes, as well as endangered breeds benefiting from conservation programs. Six different methods for computing *N*_*e*_ were compared in order to provide practical advice to breeders and stakeholders, for choosing endangerment thresholds according to species and for predicting *N*_*e*_ accurately with more or less sophisticated methods.

## Methods

### Breeds studied

Pedigree files for 60 dog, 40 sheep, 20 cattle and 20 horse breeds were extracted from French national data bases. For each species, breeds were chosen to represent a wide range of situations i.e. actual population size, endangerment status (28 populations among the sheep, cattle, and horse breeds studied have received financial support from the French government through subsidies for endangered breeds), breeding purpose (for example, selection for meat or milk), or geographical origin (local, imported or transnational populations).

In order to define the reference populations, generation intervals (*T*) were computed in the four pathways (see below), as the average age of parents when their useful offspring are born (i.e. offspring, which in turn become parents) over a 10 year period before a reference year (2005 for dog breeds (see [10]), and 2007 for sheep, cattle and horse breeds). Reference populations were defined as all the individuals (or only females for sheep and cattle breeds, given the small number of males raised in these species) with both parents known, born during a generation interval period before the reference year.

### Methods used to estimate effective population size *N*_*e*_

#### Method based on sex ratio: N_es_

Wright’s model [1] for estimating *N*_*es*_ is based on sex ratio. This very simple method is supposed to reflect the increased effects of both inbreeding and variance of progeny size under several assumptions, including random mating, no selection and random variation of progeny size across parents. Computation of *N*_*es*_ only requires the estimated numbers of breeding males (*M*) and females (*F*) in the reference population and follows equation (1):

(1) $$N_{\mathrm{es}}=\frac{4\mathrm{MF}}{M+F}.$$

#### Method based on the variance of progeny size: N_ev_

This method is more sophisticated than the previous one since it directly takes into account the observed variance of progeny size [6]. Parents of the reference population are considered as a group of useful offspring. In each pathway (*mm* = sire-sire, *mf* = sire-dam, *fm* = dam-sire or *ff* = dam-dam), observed variance (*σ*^2^) and covariance (*σ*) of progeny size are computed considering those individuals and their own parents (i.e. the grand-parents of the reference population). *N*_*ev*_ is then computed using equation (2) in which *M*_*r*_ and *F*_*r*_ are the numbers of new male and female parents beginning to reproduce each year averaged over the 10 years before the reference year:

(2) $$\begin{array}{ll} \frac{1}{N_{\mathrm{ev}}}= & \frac{1}{16M_{r}T}\left[2+\sigma_{\mathrm{mm}}^{2}+2\frac{M_{r}}{F_{r}}\sigma_{\mathrm{mm},\mathrm{mf}}+{\left(\frac{M_{r}}{F_{r}}\right)}^{2}\sigma_{\mathrm{mf}}^{2}\right]+\\ & \frac{1}{16F_{r}T}\left[2+\sigma_{\mathrm{ff}}^{2}+2\frac{F_{r}}{M_{r}}\sigma_{\mathrm{fm},\mathrm{ff}}+{\left(\frac{F_{r}}{M_{r}}\right)}^{2}\sigma_{\mathrm{fm}}^{2}\right] \end{array}.$$

#### Method based on inbreeding rate between two successive generations: N_eFt_

Considering two successive generations *t* and *t*-1, inbreeding rate (Δ*F*_*t*_) can be computed using equation (3) according to [2], in which *F*_*t+1*_ is the average coefficient of inbreeding of the reference population, and *F*_*t*_ the average coefficient of inbreeding of their parents:

(3) $$\text{Δ}F_{t}=\frac{F_{t+1}-F_{t}}{1-F_{t}}.$$

The effective population size can then be computed using the formula *N*_*eFt*_ = 1/2Δ*F*_*t*_.

#### Method based on coancestry rate between two successive generations: N_eCt_

Taking into consideration the average coefficient of coancestry (*C*), the preceding model can be applied using *C*_*t+1*_, the average coefficient of coancestry between the animals in the reference population, and *C*_*t*_, the average coefficient of coancestry between the parents of this reference population, instead of *F*_*t*+1_ and *F*_*t*_. Since the number of coancestry coefficients to be computed within a population of size *n* is equal to *n*(*n* -1)/2, computation of average coancestry can be very time-consuming in large populations. Therefore, when *n*(*n* -1)/2 is larger than 100 000, 100 000 pairs of individuals are sampled at random with *C* estimated as the mean value of the 100 000 computed coefficients.

#### Method based on individual inbreeding rate: N_eFi_

Guttiérez et al [8] proposed a method in which the level of pedigree knowledge of a given individual *i* is estimated by the number of equivalent complete generations traced (*EqG*_*i*_), computed as the sum over all known ancestors of the terms (1/2*g*), where *g* is the ancestor’s generation number, which is equal to one for the parents, two for the grandparents, etc. [17]. The approximate individual inbreeding rate Δ*F*_*i*_ is calculated according to equation (4) in which *F*_*i*_ is the coefficient of inbreeding of individual i:

(4) $$\text{Δ}F_{i}=1-\sqrt[{\mathrm{Eq}G_{i}-1}]{\left(1-F_{i}\right)}.$$

Individual inbreeding rates are averaged as $\text{Δ}\overset{―}{F}$ which leads to the following estimate of *N*_*e*_:

(5) $$N_{\mathrm{eFi}}=1/2\overset{―}{\mathrm{\Delta F}},$$

while the standard error can be approximated as:

(6) $$\sigma_{N_{\mathrm{eFi}}}=\frac{2}{\sqrt{n}}{N_{\mathrm{eFi}}}^{2}\sigma_{\text{Δ}F_{i}}$$

with *n* being the reference population size and $\sigma_{\text{Δ}F_{i}}$ the standard deviation of Δ*F*_*i*_.

#### Method based on individual coancestry rate: N_eCi_

Cervantes et al [9] proposed to approximate coancestry rate Δ*C*_*ij*_ between two individuals *i* and *j* using equation (7), in which *EqG*_*i*_ and *EqG*_*j*_ are their respective equivalent complete traced generations, and *C*_*ij*_ their coefficient of coancestry:

(7) $$\text{Δ}C_{\mathrm{ij}}=1-\sqrt[{\left(\mathrm{Eq}G_{i}+\mathrm{Eq}G_{j}\right)/2}]{\left(1-C_{\mathrm{ij}}\right)}.$$

When necessary, the coancestry over 100 000 pairs of sampled individuals was averaged, while the standard error of *N*_*eCi*_ was approximated as:

(8) $$\sigma_{N_{\mathrm{eCi}}}=\frac{2}{\sqrt{k}}{N_{\mathrm{eCi}}}^{2}\sigma_{\text{Δ}C_{\mathrm{ij}}},$$

where *k* is the number of coefficients computed (either *n*(*n* -1)/2 or 100 000) and $\sigma_{\text{Δ}C_{\mathrm{ij}}}$ is the standard deviation of Δ*C*_*ij*_.

In order to characterise the differences between average inbreeding $\overset{―}{F}$ and coancestry $\overset{―}{C}$ coefficients within reference populations, an equivalent of Nei’s fixation index *F*_*IS*_[18] was computed as follows:

(9) $$F_{\mathrm{IS}}=1-\frac{1-\overset{―}{F}}{1-\overset{―}{C}}$$

All the pedigree analyses were performed using PEDIG software [19] and our own FORTRAN routine procedures.

In order to assess the ranges of *N*_*e*_ values according to species and methods, variance was analysed using SAS software (version 9.1.3 for windows), removing the results with a negative Δ*IBD*, with REML. *N*_*e*_ was considered as the dependent variable with the following model:

(10) $$N_{\mathrm{eijk}}=\mu +\alpha_{i}+\beta_{j}+\gamma_{\mathrm{ij}}+\varepsilon_{\mathrm{ijk}},$$

where *α*_*i*_ is the computation method, *β*_*j*_ the species*,* and *γ*_*ij*_ the interaction between computation method and species, all considered as explanatory fixed factors, and *ε*_*ijk*_ is the error term following a distribution N(0,σ_*ij*_). This model was chosen because when including breed as a random effect (here, breeds are considered as samples within each species) no significant effect was observed and because the model minimizes both Akaike Information (AIC) and Bayesian Information (BIC) Criteria (see Additional file 1: Table S1).

Finally, in addition to assessing ranges of *N*_*e*_ values, we examined if ranking was similar with the different methods by performing for each species, a Principal Component Analysis (PCA), considering breeds as observations and the six methods as variables.

## Results and discussion

### Demographic and genealogical parameters

Depending on breed and species, reference population sizes ranged from 110 (Barbet dog breed) to 2 122 041 (Holstein cattle breed). Only six cattle, one sheep and one horse breeds had a reference population size larger than 100 000 and only one cattle, six sheep, five horse and six dog breeds had a reference population size smaller than 1000 [see Additional file 2: Table S1 to S4]. The timeframe used to constitute the reference populations was computed on the basis of the average generation interval *T* for each species. The mean value of *T* across breeds varied among species (Table 1). In particular, horse breeds had quite larger *T* values than cattle, sheep or dog breeds (T-test, *P* < 0.001) whereas the level of pedigree knowledge was lower for horses than for the other species with an average *EqG* equal to 4.4 (*P* < 0.001) compared to 6.1, 6.0 and 5.8 in cattle, sheep and dog, respectively. This difference may be explained by the high level of crossbreeding in horse populations, since the pedigree files chosen here were restricted to purebred animals.

**Table 1:** Genealogical parameters and effective population sizes for the 140 breeds studied averaged for each species

					**IBD methods**					
**Species**	**Breed nb**	***T***	**E*qG***	***F***_***IS***_**%**	***N***_***eCi***_	***N***_***eCt***_	***N***_***eFi***_	***N***_***eFt***_	***N***_***es***_	***N***_***ev***_
Cattle	20	5.4 [4-7.2]	6.1 [3.4-8.3]	-0.45 [-1.87-1.44]	245 [55-958]	91 [27-242]	182 [58-646]	100 [35-204]	21,648 [208-133056]	934 [108-4420]
Sheep	40	3.6 [2.9-4.1]	6.0 [2.6-10.3]	-0.37 [-4.28-2.44]	189 [28-429]	68 [18-142]	191 [38-675]	95 [21-375]	1502 [30-13736]	407 [46-1812]
Horse	20	9.6 [6.8-13.7]	4.4 [1.8-7.6]	-0.1 [-1.98-2.39]	184 [33-520]	175 [44-799]	135 [22-321]	125 [33-257]	1906 [111-6349]	487 [53-2022]
Dog	60	4.1 [2.7-5.1]	5.8 [3-9.2]	1.37 [-2.87-4.7]	204 [21-692]	241 [17-1451]	89 [22-392]	80 [15-510]	1472 [37-6041]	471 [35-1443]
Total	140	4.9 [2.7-13.7]	5.7 [1.8-10.3]	0.41 [-4.28-4.7]	203 [21-958]	160 [17-1451]	138 [22-675]	93 [15-510]	4425 [30-133056]	356 [35-4420]

IBD = identity by descent; nb = number; *T *= average generation length in years; *EqG *= number of equivalent generations; *F*_*IS *_= fixation index; *N*_*eCi *_= method based on individual coancestry rate; *N*_*eCt *_= method based on coancestry rate between two successive generations; *N*_*eFi *_= method based on individual inbreeding rate; *N*_*eFt *_= method based on inbreeding rate between two successive generations ; *N*_*es *_= *N*_*e *_method based on sex ratio; *N*_*ev *_= method based on variance of progeny size; in brackets, minimal and maximal values.

Similar to the heterogeneity of pedigree knowledge, average IBD coefficients (average *C* and *F*) ranged from 0.2% (*C* and *F* in Comtois horse breed) to 9.1% (*C* in the Barbet dog breed). Pearson correlations between *EqG* and IBD coefficients were equal to 0.45 and 0.23 for *F* and *C*, respectively, while they were larger (*r* = 0.67) between *F* and *C* [see Additional file 3: Figures S1, S2, S3]. Differences between *C* and *F*, measured by the fixation index *F*_*IS*_, varied more or less according to species. Average *F*_*IS*_ values were negative in cattle, sheep and horse breeds i.e. -0.45%, -0.37%, -0.1%, respectively and positive i.e. 1.37% (with *P* < 0.001) in dog breeds, underlining the existence of population substructure within most dog breeds.

### Variance analysis of effective population size estimates

Depending on breed, species and method, *N*_*e*_ values varied greatly i.e. between 15 (*N*_*eFt*_ for the Saarloos Wolfdog breed) and 133 056 (*N*_*es*_ for the Charolais cattle breed). When IBD rate was negative i.e. indicating a decrease in *C* or *F* between the last two generations, *N*_*eFt*_ (four breeds) and *N*_*eCt*_ (one breed) were not calculated.

**Figure 1:**
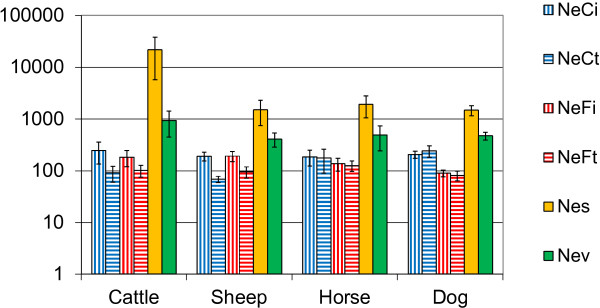
**Average effective population sizes according to species and methods, using a logarithmic scale. **Standard errors are indicated.

Effects of “computation method” and “interaction of computation method x species” on *N*_*e*_ were significant (*P* < 0.0001) unlike that of “species” only (*P* = 0.06). Indeed, the different models applied produced contrasted results (Figure 1 and Table 1) i.e. *N*_*e*_ estimates were much larger with *N*_*es*_ (4425 on average) and, to a lesser extent, with *N*_*ev*_ (356 on average) than with *N*_*e****Ft***_ (93), *N*_*eFi*_ (138), *N*_*eCt*_ (160) and *N*_*eCi*_ (203), although some differences were observed among these last four methods. In relation to the positive *F*_*IS*_ values obtained for dog breeds, *N*_*e*_ values were higher with the two methods based on coancestry *C* (*N*_*eCi*_ = 204 and *N*_*eCt*_ = 241 on average) compared to those based on inbreeding *F* (*N*_*eFi*_ = 89 and *N*_*e****Ft***_ = 80 on average) (*P* < 0.0001). Such a significant difference was not observed for the other species. As illustrated in Table S5 of Additional file 2, residual standard deviations varied with “computation method” and “species” i.e. for IBD methods: they ranged from 54 (*N*_*eFi*_ for dog) to 253 (*N*_*eCi*_ for cattle); for *N*_*es*_, they reached 36 268 for cattle. This result justified the computation of an error component specific for “computation method” and “species”. With the methods based on IBD rate, within-population standard errors (s.e.) ranged from 0.1 to 10.1 for *N*_*eCi*_ and from 0 to 172.9 for *N*_*eFi*_. Among the 140 breeds studied here, s.e. mean values were equal to 1.3 and 7.0 for *N*_*eCi*_ and *N*_*eFi*_, respectively (s.d. mean values across breeds were equal to 1.6 and 20.0, respectively; results not shown).

### Principal component analysis of effective population size estimates

Results of the principal component analysis showed that the two main components explained between 74% (dog) and 87% (cattle) of the total inertia depending on the species considered. Two tendencies were observed. For cattle, each variable was highly correlated with the first component (*r* > 0.72) [see Additional file 4], while for dog, *N*_*eFi*_ and *N*_*e****Ft***_ were correlated weakly with the first component (*r* = 0.03 and 0.22, respectively) but highly with the second component (*r* = 0.80 for both variables). For sheep and horse, intermediate values were obtained. These results agree with the Kendall correlations computed between each method (Table 2). For dog, *N*_*eFi*_ and *N*_*e****Ft***_ were moderately correlated with the other estimates (below 0.36 compared to above 0.45 in all other cases). From a general point of view, *N*_*eCi*_ was highly correlated with the other estimates (0.52 on average) while *N*_*e****Ft***_ was moderately correlated (0.33 on average).

**Table 2:** **Kendall correlations between methods used to estimate *N***_***e***_**for each species**

Sheep		**Cattle**	**IBD methods**					
			***N***_***eCi***_	***N***_***eCt***_	***N***_***eFi***_	***N***_***eFt***_	***N***_***es***_	***N***_***ev***_
IBD methods	*Ne*_*Ci*_			**0.77**	**0.75**	**0.55**	**0.44**	**0.72**
	*Ne*_*Ct*_		0.48		**0.64**	**0.61**	**0.32**	**0.59**
	*Ne*_*Fi*_		0.70	0.50		**0.65**	**0.36**	**0.51**
	*Ne*_***Ft***_		0.31	0.45	0.50		**0.16**	**0.33**

	*Ne*_*s*_		0.60	0.23	0.41	0.09		**0.62**
	*Ne*_*v*_		0.70	0.28	0.52	0.13	0.77	
Dog		**Horse**	**IBD methods**					
			***N***_***eCi***_	***N***_***eCt***_	***N***_***eFi***_	***N***_***eFt***_	***N***_***es***_	***N***_***ev***_
IBD methods	*Ne*_*Ci*_			**0.53**	**0.33**	**0.23**	**0.53**	**0.74**
	*Ne*_*Ct*_		0.63		**0.17**	**0.31**	**0.54**	**0.49**
	*Ne*_*Fi*_		0.29	0.36		**0.61**	**0.08**	**0.13**
	*Ne*_Ft_		0.17	0.36	0.55		**0.11**	**0.10**

	*Ne*_*s*_		0.45	0.58	0.19	0.27		**0.71**
	*Ne*_*v*_		0.59	0.50	0.12	0.16	0.68	

IBD = identity by descent; *N*_*eCi *_= method based on individual coancestry rate; *N*_*eCt *_= method based on coancestry rate between two successive generations; *N*_*eFi *_= method based on individual inbreeding rate; *N*_*eFt *_= method based on inbreeding rate between two successive generations; *N*_*es *_= *N*_*e *_method based on sex ratio; *N*_*ev *_= method based on variance of progeny size.

**Figure 2:**
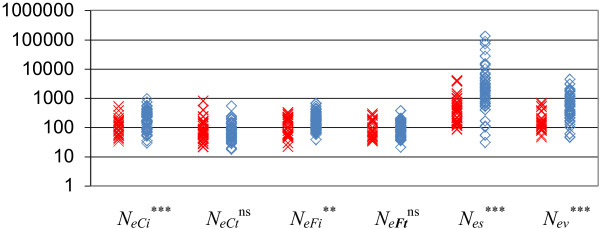
**Effective population size of cattle, horse, and sheep breeds, using a logarithmic scale. **red X = breeds receiving endangered breed subsidies; blue ◊ = other breeds; difference in ranking between both categories using the Wilcoxon test: ns non significant, ** P < 0.01, *** P < 0.001.

Figure 2 illustrates the differences in *N*_*e*_ values for the different methods according to whether a breed is receiving or not financial support because of its endangered status (this is true for some cattle, horse and sheep breeds but not for rare dog breeds, which do not receive public financial help). With models *N*_*e****Ct***_ and *N*_*e****Ft***_, *N*_*e*_ rankings did not differ significantly between endangered and non-endangered breeds. With the other models, *N*_*e*_ values (*P* < 0.01) were lower for endangered than non-endangered breeds although they were not entirely discriminated.

## Discussion

This study allowed us to analyse the specificities of each of the four included species with regards to the assessment of their effective population size estimated with different approaches.

Genealogical parameters were quite similar to previously reported results [10-13,20-27], although for horse, pedigree knowledge was relatively low, because horse breeds’ pedigree were restricted to individuals belonging to each breed. We would also like to underline that the Pearson correlations between *EqG* and IBD estimators were moderate, indicating that the regression suggested by Nagy et al. [26] between pedigree knowledge and IBD is not straightforward.

The effective population sizes computed here were on average of the same magnitude as those reported in other studies using similar approaches for cattle [14,20,21], sheep [22,23], or horse [11,27]. For dog, previous studies [10,24-26] applied inbreeding approaches to compute *N*_*e*_, with average values close to 100 (ranging from 17 to 1090), which is in agreement with our results.

In this data set, the largest populations concerned cattle as expected, given the high level of homogenization in this species due to intense selection. For instance, in France, out of 46 different cattle breeds, the main five breeds (namely, Holstein, Charolais, Limousine, Montbéliarde and Blonde d’Aquitaine) account for 80% of the total cattle stock (estimated to be 8 million cows; source: France Génétique Elevage, http://www.france-genetique-elevage.fr/). Among the six methods used to compute *N*_*e*_ for cattle populations, those based on sex-ratio (*N*_*es*_) and those taking into account variance of progeny size (*N*_*ev*_) or directly measuring IBD increase produced very different results (Figure 1). This is explained by the wide use of artificial insemination (AI) in cattle (particularly in dairy cattle) with a small number of sires producing thousands of offspring, although cattle have a low prolificacy compared to dogs. Such a contrast was not observed for sheep because (among other reasons) AI is not as developed in sheep as in cattle and a ram cannot provide as many doses as a bull. For dog, the most striking result was the difference between methods based on coancestry *C* and those on inbreeding *F* evolutions, which is linked to the positive *F*_*IS*_ values found for this species. Under panmixia, both *C* and *F* parameters are assumed to differ only by **Δ***IBD*, the average coancestry of reproducers corresponding to the average inbreeding of the next generation. This is why, in random mating conditions at least, it is expected that *C* is larger than *F*, and thus that *F*_*IS*_ is negative. This was not the case for most of the dog breeds (and some breeds of the other species) either because of the existence of subpopulations or of particular breeding practices such as a high frequency of mating between close relatives [28]. As a consequence, when *F* was used instead of *C* to compute *N*_*e*_, on average, *N*_*e*_ was divided by more than two in dogs. Indeed, it has been shown that if inbreeding is used as an estimator of population genetic diversity bias can occur because of population substructure [11,29]. Such phenomena are often observed for dog breeds. Since all previous reports on *N*_*e*_ of dog breeds were based on *F* coefficients, they must be largely underestimated. From a more general point of view, for a domestic or captive population with more or less substructure, the method based on coancestry is the most appropriate to compute *N*_*e*_.

Table 3 lists the factors and assumptions that distinguish the six genealogical methods that were applied to compute *N*_*e*_. First, these methods measured different parameters; some methods used demographic parameters [6] to assess variance in allele frequencies and increase in inbreeding, i.e. number of reproducers for *N*_*es*_ and variance of their progeny size for *N*_*ev*_, whereas other methods used coancestry or inbreeding rate to measure the evolution of IBD probability directly. In addition, estimation of these rates differed with the method used, which, among other consequences, impacted the time scale considered. The advantage of methods based on IBD increases between successive generations (*N*_*eCt*_ and *N*_*eFt*_) is the possibility of choosing the time length included in the computation model and thus analysing the evolution of IBD probability during a variable number of years or generations. However, they also have several weaknesses that are related with the fluctuation of IBD over time because of changes in breeding schemes, registration of individuals without knowing their pedigree or sampling effects. Indeed, IBD can decrease over a given period, leading to a negative *N*_*e*_ value. This is a problem, particularly when analysing a large number of breeds in which case, determining a time period during which IBD does not decrease in any of the breeds is almost impossible. Here, we chose a relatively short period of time (two generations) to estimate *N*_*eCt*_ and *N*_*eFt*_, and among the 280 estimations, negative values were observed in five cases only. In the literature, studies considering longer periods of time to compute *N*_*e*_ encountered the same problem even for a more or less small number of breeds [10,12,27]. Methods based on individual IBD probabilities (*N*_*eCi*_ and *N*_*eFi*_) clearly overcome this problem, since the computation is based on the rooting of IBD coefficients by *EqG*. With these methods, knowledge of the whole pedigree is taken into account. However, this means that for breeds with different levels of pedigree knowledge, the time period considered will vary according to breed. Another difference in these methods is the sample considered and therefore the precision of *N*_*e*_ estimation. Since coancestry is averaged on a much larger number of coefficients than inbreeding (see Table 3), the precision of *N*_*e*_ estimation is expected to be higher in the first case, as underlined by Cervantes et al. [9]. For breeds with large current population sizes, it may be necessary to average coancestry on a sample of individual pairs (100 000 in our case) to overcome the problem of computing time. Even in such situations, standard error was on average five times lower with coancestry than with inbreeding.

**Table 3:** **Characteristics of the different methods used to compute effective population size *N***_***e***_

**Method**	**Genealogy required**	**Parameters measured**	**Indicator used to compute *N***_***e***_	**Time period or number of generations taken into account**	**Theoretical sample size for a reference population of size n**
*N*_*es*_	no	change in allele frequency / heterozygosity loss	number of reproducers	generation n	-
*N*_*ev*_	yes	change in allele frequency / heterozygosity loss	variance/covariance of progeny sizes	generation n-1	-
*N*_*eFt*_	yes	heterozygosity loss	inbreeding	period or number of generations to be fixed	n
*N*_*eCt*_	yes	heterozygosity loss	coancestry	period or number of generations to be fixed	n x (n-1)
*N*_*eFi*_	yes	heterozygosity loss	inbreeding	all known generations	n
*N*_*eCi*_	yes	heterozygosity loss	coancestry	all known generations	n x (n-1)

*N*_*eCi*_ = method based on individual coancestry rate; *N*_*eCt*_ = method based on coancestry rate between two successive generations; *N*_*eFi*_ = method based on individual inbreeding rate; *N*_*eFt*_ = method based on inbreeding rate between two successive generations; *N*_*es*_ = *N*_*e*_ method based on sex ratio; *N*_*ev*_ = method based on variance of progeny size.

The issue of minimum viable population sizes is not new and it has been suggested to use *N*_*e*_ thresholds of 50 and 500 for risks of extinction on the short or long runs, respectively [4]. Although the existence of these “magic numbers” has been discussed and criticized, they do constitute an interesting tool for stakeholders [30]. According to the FAO [31], a breed can be categorized as critical if the total number of breeding females is less or equal to 100 or the total number of breeding males is less or equal to 5, and endangered if the total number of breeding females is less or equal to 1000 or the total number of breeding males is less or equal to 20. Since pedigree information is not always available, i.e. for livestock breeds in developing countries or wild populations, the FAO has based its recommendations on sex ratio considerations (similar to those in the *N*_*es*_ computation) to determine the level of endangerment of a breed. However, as underlined in our study and by Martyniuk [32], the FAO figures for breed risk-status do not provide a full picture of the level of genetic diversity.

Given the contrasted results obtained for cattle between the *N*_*es*_ and the more sophisticated methods, we recommend choosing a higher threshold when considering endangerment level of cattle in comparison to other species, at least in breeds in which animals are mainly bred via AI. Comparing rankings of *N*_*e*_ estimated with the method based on sex-ratio and the more sophisticated ones showed interesting results. In the comparison with the *N*_*eCi*_ method, which does not suffer from bias linked to population substructure, sampling size or IBD decrease, the correlation ranged among species from 0.44 (cattle) to 0.60 (sheep). By contrast, correlations between *N*_*ev*_ that takes variance of progeny size into account and *N*_*eCi*_ were much larger and ranged ranging from 0.59 (dog) to 0.74 (horse). This indicates that, even if the number of reproducing males and females is a major explanatory factor for variation in effective population size, other parameters and, in particular, unbalanced progeny sizes may differ greatly according to breeds. Thus, caution must be taken when interpreting estimated effective population sizes.

According to the French law, a breed may receive financial support as an endangered breed, if it is considered as a French indigenous population and if the total number of females is below a threshold defined - by species - by the European Union (European Union Commission Regulation 445/2002 and 817/2002). As an example, the Clun Forest or the Finnish sheep breeds are not considered as endangered since they are not French. This explains why even if *N*_*e*_ is estimated with the method based on demographic parameters (*N*_*es*_), some breeds receive financial support although they have a larger *N*_*e*_ than others which do not receive support. This discrepancy is even more pronounced with other methods that take into account other parameters impacting effective population size (Figure 2).

Among other methods to measure effective population size, molecular approaches may constitute an interesting option, especially if many markers are available. Indeed, methods based on linkage disequilibrium may provide interesting and original information since they can estimate the evolution of effective population size over former generations [33]. When computing effective population size for the international Holstein breed, using between 3000 and 10 000 SNP and the linkage disequilibrium approach, de Roos et al. [34] reported *N*_*e*_ values ranging from 64 to 90 according to country, which are of the same order of magnitude as those calculated in our study with the most sophisticated methods *N*_*eCi*_ = 93 and *N*_*eFi*_ = 91. However, it should be underlined that similar to the pedigree-based methods, the different molecular methods may give divergent results depending on the sampling strategy or the parameter used to compute *N*_*e*_ (evolution of heterozygosity or variance of allele frequency over time, linkage disequilibrium,…) [35,36]. Moreover, given the cost of genotyping, pedigree knowledge will continue to represent a valuable information source in the coming years in many cases.

## Conclusions

In this study, we show that indicators of effective population size may follow different trends depending on the species studied and, in particular, on the genetic structure existing within the breed. Further studies are necessary to improve the accuracy of genealogical methods, for instance taking better account of heterogeneity in pedigree knowledge. Finally, it must be stated, that for conservation issues, socio-cultural background is at least as important as effective population size, and should, when possible, be taken into account when assessing the endangerment level of a given breed (e.g., [37]).

## Competing interests

The authors declare that they have no competing interests.

## Authors’ contributions

GL and EV jointly conceived the design of the study and discussed the results. GL, CDB and SD extracted the pedigree data for dog, cattle and sheep, and horse, respectively. GL computed the pedigree estimators. TMH performed the statistical analysis. GL wrote the first draft of the manuscript, which was then modified and discussed with co-authors. All authors read and approved the final manuscript.

## Supplementary Material

Additional file 1 **Model selection. **This file contains information about models tested for variance analysis of effective population size estimates. Click here for file

Additional file 2 **Genealogical parameters and effective population sizes for the 20 cattle breeds.** The tables S1 to S4 provide genealogical parameters and *N*_*e *_estimates for each breed species by species. Table S5 provides residual standard deviations according to methods and species after variance analysis of effective population size estimates. Click here for file

Additional file 3 **Relation between equivalent complete generations traced *EqG *and inbreeding *F *(Figure S1), *EqG *and coancestry *C *(Figure S2), *F *and *C *(Figure S3) for the 140 breeds. **Figures S1, S2 and S3 show relations between genealogical indicators according to species studied. Click here for file

Additional file 4 **Correlation circles from the principal component analysis considering the six effective population sizes computed for the four species independently.** This figure shows correlations circle between computation methods and the two first components, after PCA have been independently performed for each of the four species. Click here for file
