# Supplementary material for: Methods to estimate effective population size using pedigree data: Examples in dog, sheep, cattle and horse
Source: Genet Sel Evol. 2013 Jan 2;45(1):1. doi: 10.1186/1297-9686-45-1 (PMC3599586; doi:10.1186/1297-9686-45-1)
Supplement: Additional file 1 — Model selection. This file contains information about models tested for variance analysis of effective population size estimates. [file 1297-9686-45-1-S1.docx]

**Supplementary Document 1: Model selection**

Let *N_eijk_* be the effective size of breed *k* in species *j* estimated with computation method

*i*. To analyze the data and quantify the effect of computation method, species and their interaction on the response variable *N_e_*, we considered several (mixed) models, all of the form

$$N_{eijk}=\mu+\alpha_{i}+\beta_{j}+\gamma_{ij}+U_{k(j)}+\varepsilon_{ijk}$$

where *α_i_, β_j_* and *γ_ij_* have the same definition as in the article, and random effect *U_k(j)_* stands for the (random) effect of population *k* in specie *j*. All models have the same list of fixed effects, and differ only through the modeling of the variances of the random effects *U_k(j)_* and *$\varepsilon$_ijk_* . The following cases have been considered:

1. V(*U_k(j)_*) = *σ_U_²* , V(*ε_ijk_*) = *σ²*,
2. V(*U_k(j)_*) = *σ_j_²* , V(*ε_ijk_*) = *σ²*,
3. V(*U_k(j)_*) = 0 (no random effect U) , V(*ε_ijk_*) = *σ_i_²*,
4. V(*U_k(j)_*) = *σ_U_²* , V(*ε_ijk_*) = *σ_i_²*,
5. V(*U_k(j)_*) = *σ_j_²* , V(*ε_ijk_*) = *σ_i_²*,
6. V(*U_k(j)_*) = 0 , V(*ε_ijk_*) = *σ_ij_²*,

All these models were compared using the AIC and BIC criteria to select the best variance modeling (for these two criteria the "smaller is better" rule applies). Results are displayed here. Model (6) that was used for the analyses displayed in the article clearly outperforms its competitors.

| Model | AIC | BIC |
| --- | --- | --- |
| (1) | 16387.0 | 16392.8 |
| (2) | 16293.0 | 16298.9 |
| (3) | 11835.1 | 11863.5 |
| (4) | 15975.2 | 15995.8 |
| (5) | 15996.9 | 16017.5 |
| (6) | 11148.5 | 11262.1 |

The different models and their AIC and BIC evaluations.
