# Supplementary material for: Methods to estimate effective population size using pedigree data: Examples in dog, sheep, cattle and horse
Source: Genet Sel Evol. 2013 Jan 2;45(1):1. doi: 10.1186/1297-9686-45-1 (PMC3599586; doi:10.1186/1297-9686-45-1)
Supplement: Additional file 2 — Genealogical parameters and effective population sizes for the 20 cattle breeds. The tables S1 to S4 provide genealogical parameters and Ne estimates for each breed species by species. Table S5 provides residual standard deviations according to methods and species after variance analysis of effective population size estimates. [file 1297-9686-45-1-S2.doc]

Table S1: Genealogical parameters and effective population sizes for the 20 cattle breeds

| Breed | Type | *Pref* | *T* | *EqG* | *C* (%) | *F* (%) | *F_IS_*  (%) | *IBD* | | | | *N_es_* | *N_ev_* |
| --- | --- | --- | --- | --- | --- | --- | --- | --- | --- | --- | --- | --- | --- |
|  |  |  |  |  |  |  |  | *N_eCi_* | *N_eCt_* | *N_eFi_* | *N_eCt_* |  |  |
| Abondance | dairy | 24218 | 5.2 | 5.4 | 5.2 | 3.6 | -1.68 | 52 | 19 | 68 | 35 | 2346 | 255 |
| Aubrac | meat | 78285 | 5.3 | 7.8 | 0.9 | 1.2 | 0.29 | 443 | 227 | 291 | 180 | 15262 | 2060 |
| Bazadaise^x^ | meat | 4350 | 6.3 | 5.8 | 1.9 | 2.4 | 0.47 | 153 | 77 | 104 | 67 | 966 | 512 |
| Blonde d'Aquitaine | meat | 282534 | 5.2 | 5.8 | 1.3 | 1.4 | 0.05 | 227 | 61 | 194 | 86 | 45809 | 941 |
| Brown-Swiss | dairy | 25361 | 5.4 | 5.7 | 3.4 | 2.6 | -0.83 | 85 | 55 | 98 | 80 | 2623 | 396 |
| Charolaise | meat | 773375 | 4.8 | 7.9 | 0.4 | 0.5 | 0.11 | 958 | 198 | 646 | 200 | 133056 | 4420 |
| Ferrandaise^x^ | meat | 587 | 7.2 | 3.7 | 3.4 | 2.8 | -0.70 | 55 | 34 | 58 | 71 | 208 | 135 |
| Flamande^x^ | meat | 3442 | 7.0 | 5.8 | 3.8 | 2.7 | -1.18 | 75 | 29 | 97 | 53 | 505 | 153 |
| Gasconne | meat | 21278 | 6.1 | 3.4 | 0.3 | 0.8 | 0.53 | 562 | 242 | 139 | 82 | 4097 | 1150 |
| Limousine | meat | 419159 | 5.1 | 6.7 | 0.5 | 0.7 | 0.21 | 740 | 185 | 412 | 168 | 72540 | 1675 |
| Montbéliarde | dairy | 465255 | 4.2 | 7.1 | 4.8 | 3.8 | -1.09 | 72 | 30 | 82 | 43 | 27324 | 359 |
| Normande | dairy | 345897 | 4.2 | 8.3 | 5.1 | 4.0 | -1.12 | 80 | 37 | 93 | 47 | 10114 | 267 |
| Parthenaise | meat | 31578 | 4.6 | 6.2 | 1.1 | 1.0 | -0.03 | 295 | 133 | 263 | 157 | 4981 | 583 |
| French Red Pied | dairy | 13882 | 5.1 | 5.8 | 1.8 | 1.1 | -0.76 | 158 | 80 | 238 | 204 | 1011 | 356 |
| Holstein | dairy | 2122041 | 4.0 | 7.9 | 4.2 | 3.8 | -0.38 | 93 | 49 | 91 | 64 | 89112 | 961 |
| Rouge des prés | meat | 35511 | 4.8 | 4.8 | 0.9 | 0.7 | -0.11 | 299 | 104 | 294 | 149 | 5038 | 772 |
| Salers | meat | 66679 | 5.3 | 8.0 | 1.3 | 2.7 | 1.44 | 323 | 82 | 136 | 51 | 13734 | 3036 |
| French Simmental | dairy | 25884 | 5.9 | 5.3 | 2.3 | 1.4 | -0.97 | 114 | 110 | 167 | 169 | 2609 | 342 |
| Tarentaise | dairy | 9672 | 5.8 | 5.4 | 4.7 | 3.3 | -1.47 | 57 | 35 | 70 | 50 | 1105 | 205 |
| Vosgienne^x^ | dairy | 3899 | 6.0 | 4.7 | 3.8 | 2.0 | -1.87 | 60 | 27 | 101 | 51 | 516 | 108 |

*Pref,* reference population size; *T*, average generation length in years; *EqG*, number of equivalent generations; *F_IS_*, fixation index; *N_eCi_*, *N_e_* based on individual coancestry rate model; *N_eCt_*, *N_e_* based on coancestry rate between two successive generations model; *N_eFi_*, *N_e_* based on individual inbreeding rate model; *N_eFt_*, *N_e_* based on inbreeding rate between two successive generations model; *N_es_*, *N_e_* based on sex ratio based model; *N_ev_*, *N_e_* based on variance of progeny size model; ^x^ breeds receiving endangered breed subsidies.

Table S2: Genealogical parameters and effective population sizes for the 40 sheep breeds

| Breed | Type | *Pref* | *T* | *EqG* | *C* (%) | *F* (%) | *F_IS_*  (%) | *IBD* | | | | *N_es_* | *N_ev_* |
| --- | --- | --- | --- | --- | --- | --- | --- | --- | --- | --- | --- | --- | --- |
|  |  |  |  |  |  |  |  | *N_eCi_* | *N_eCt_* | *N_eFi_* | *N_eFt_* |  |  |
| Avranchin^x^ | meat | 412 | 3.3 | 4.4 | 4.0 | 3.3 | -0.68 | 55 | 40 | 52 | 48 | 129 | 77 |
| Basco-bearnaise | dairy | 13871 | 3.6 | 5.8 | 3.1 | 2.4 | -0.67 | 95 | 33 | 109 | 48 | 1639 | 348 |
| Berrichon de l'Indre^x^ | meat | 1204 | 3.5 | 2.6 | 3.3 | 1.6 | -1.73 | 38 | 21 | 54 | 35 | 86 | 47 |
| Berrichon du Cher | meat | 9697 | 3.9 | 8.9 | 4.6 | 4.3 | -0.30 | 94 | 44 | 89 | 50 | 901 | 155 |
| Bizet^x^ | meat | 6002 | 3.8 | 4.2 | 1.4 | 1.0 | -0.37 | 154 | 66 | 174 | 257 | 437 | 208 |
| Blanc du Massif Central | meat | 32960 | 3.7 | 5.3 | 0.7 | 0.7 | -0.02 | 398 | 97 | 356 | 108 | 2276 | 1205 |
| Bleu du Maine^x^ | meat | 2153 | 3.2 | 8.0 | 2.2 | 1.9 | -0.32 | 177 | 142 | 201 | 293 | 359 | 162 |
| Boulonnais^x^ | meat | 1283 | 3.4 | 2.7 | 1.4 | 0.8 | -0.55 | 100 | 41 | 95 | 80 | 219 | 113 |
| Causse du lot | meat | 8593 | 3.7 | 4.5 | 1.0 | 0.6 | -0.33 | 240 | 61 | 323 | 101 | 391 | 341 |
| Charmoise^x^ | meat | 6972 | 3.5 | 6.8 | 2.1 | 2.0 | -0.13 | 163 | 64 | 154 | 77 | 980 | 352 |
| Charollais | meat | 32207 | 2.9 | 9.6 | 1.6 | 1.9 | 0.30 | 297 | 86 | 224 | 85 | 3907 | 1118 |
| Clun forest | meat | 357 | 3.9 | 3.1 | 5.1 | 1.5 | -3.85 | 28 | 44 | 76 | 85 | 30 | 47 |
| Corse | dairy | 6406 | 3.8 | 3.0 | 0.7 | 0.2 | -0.49 | 220 | 95 | 675 | 375 | 778 | 216 |
| Cotentin^x^ | meat | 426 | 3.2 | 4.7 | 2.3 | 2.5 | 0.25 | 99 | 45 | 62 | 38 | 168 | 86 |
| Dorset | meat | 795 | 3.6 | 6.4 | 4.7 | 7.0 | 2.44 | 68 | 36 | 38 | 21 | 165 | 102 |
| Est à laine merinos | meat | 4574 | 4.1 | 4.3 | 1.2 | 1.0 | -0.27 | 179 | 65 | 175 | 86 | 518 | 165 |
| Finnish | meat | 256 | 3.8 | 6.1 | 8.4 | 4.5 | -4.28 | 35 | 52 | 56 | 96 | 54 | 46 |
| Grivette | meat | 7982 | 3.5 | 4.7 | 0.9 | 0.6 | -0.33 | 256 | 87 | 330 | 112 | 651 | 262 |
| Hampshire | meat | 2843 | 3.6 | 6.1 | 2.1 | 2.7 | 0.58 | 145 | 81 | 101 | 68 | 404 | 210 |
| Ile de France | meat | 48967 | 3.7 | 8.4 | 1.0 | 1.7 | 0.68 | 424 | 75 | 223 | 55 | 4871 | 812 |
| Lacaune dairy | dairy | 441998 | 3.2 | 9.3 | 1.4 | 1.8 | 0.46 | 348 | 79 | 252 | 66 | 13736 | 1812 |
| Lacaune Viande | meat | 39068 | 3.4 | 7.2 | 1.2 | 1.6 | 0.42 | 301 | 85 | 198 | 73 | 2072 | 639 |
| Limousin | meat | 11231 | 3.7 | 7.4 | 2.4 | 2.9 | 0.52 | 157 | 41 | 120 | 37 | 696 | 349 |
| Manech tête noire | dairy | 9574 | 4.1 | 5.3 | 3.7 | 2.8 | -0.91 | 72 | 28 | 83 | 38 | 1279 | 264 |
| Manech tête rousse | dairy | 64139 | 3.4 | 6.1 | 1.9 | 1.6 | -0.28 | 167 | 56 | 172 | 77 | 7226 | 775 |
| Merinos d'Arles | meat | 6240 | 4.0 | 3.3 | 0.4 | 0.3 | -0.08 | 429 | 140 | 536 | 182 | 652 | 598 |
| Merinos de Rambouillet^x^ | meat | 247 | 3.7 | 6.4 | 3.7 | 1.3 | -2.45 | 85 | 78 | 202 |  | 115 | 93 |
| Noir du Velay | meat | 8034 | 3.4 | 3.9 | 0.7 | 0.4 | -0.36 | 259 | 94 | 446 | 192 | 559 | 312 |
| Prealpes | meat | 13189 | 3.6 | 5.3 | 1.2 | 1.2 | -0.01 | 221 | 51 | 195 | 55 | 1224 | 509 |
| Rava | meat | 14915 | 3.8 | 5.4 | 1.6 | 1.4 | -0.13 | 174 | 66 | 157 | 76 | 643 | 261 |
| Romane | meat | 22415 | 3.4 | 6.0 | 0.9 | 0.8 | -0.05 | 358 | 81 | 347 | 101 | 1806 | 584 |
| Romanov | meat | 1291 | 3.9 | 6.1 | 5.9 | 3.8 | -2.22 | 50 | 18 | 68 | 43 | 103 | 125 |
| Rouge de l'Ouest | meat | 18396 | 3.5 | 9.2 | 1.7 | 1.7 | -0.04 | 263 | 61 | 249 | 85 | 1837 | 503 |
| Roussin de la Hague^x^ | meat | 4191 | 3.1 | 5.9 | 2.7 | 2.4 | -0.35 | 108 | 68 | 106 | 163 | 734 | 190 |
| Solognote^x^ | meat | 1846 | 3.5 | 5.5 | 2.0 | 1.8 | -0.13 | 144 | 80 | 131 | 72 | 300 | 143 |
| Southdown^x^ | meat | 1088 | 3.8 | 7.4 | 5.9 | 5.4 | -0.52 | 61 | 109 | 60 | 42 | 141 | 80 |
| Suffolk | meat | 11123 | 3.6 | 6.8 | 1.1 | 1.6 | 0.46 | 310 | 105 | 184 | 69 | 1839 | 560 |
| Tarasconaise | meat | 3803 | 3.7 | 3.5 | 1.2 | 1.0 | -0.14 | 155 | 45 | 160 | 55 | 393 | 298 |
| Texel | meat | 22176 | 3.1 | 9.0 | 1.2 | 1.9 | 0.69 | 391 | 108 | 215 | 82 | 3159 | 1368 |
| Vendéen | meat | 23742 | 3.3 | 10.3 | 2.2 | 2.4 | 0.27 | 237 | 65 | 193 | 76 | 2626 | 730 |

*Pref,* reference population size; *T*, average generation length in years; *EqG*, number of equivalent generations; *F_IS_*, fixation index; *N_eCi_*, *N_e_* based on individual coancestry rate model; *N_eCt_*, *N_e_* based on coancestry rate between two successive generations model; *N_eFi_*, *N_e_* based on individual inbreeding rate model; *N_eFt_*, *N_e_* based on inbreeding rate between two successive generations model; *N_es_*, *N_e_* based on sex ratio based model; *N_ev_*, *N_e_* based on variance of progeny size model; ^x^ breeds receiving endangered breed subsidies.

Table S3: Genealogical parameters and effective population sizes for the 20 horse breeds

| Breed | Type | *Pref* | *T* | *EqG* | *C* (%) | *F* (%) | *F_IS_*  (%) | *IBD* | | | | *N_es_* | *N_ev_* |
| --- | --- | --- | --- | --- | --- | --- | --- | --- | --- | --- | --- | --- | --- |
|  |  |  |  |  |  |  |  | *N_eCi_* | *N_eCt_* | *N_eFi_* | *N_eFt_* |  |  |
| Anglo-Arab | W | 9948 | 12.8 | 4.0 | 1.2 | 1.1 | -0.06 | 170 | 81 | 141 | 83 | 1471 | 282 |
| Arab | W | 16013 | 10.7 | 6.9 | 1.6 | 3.4 | 1.89 | 221 | 155 | 84 |  | 6349 | 1343 |
| Ardennais^x^ | D | 5802 | 8.0 | 4.3 | 0.6 | 0.7 | 0.17 | 375 | 233 | 225 | 151 | 1431 | 601 |
| Auxois^x^ | D | 528 | 7.6 | 1.8 | 1.1 | 0.3 | -0.85 | 77 | 57 | 219 | 173 | 165 | 88 |
| Boulonnais^x^ | D | 2960 | 9.1 | 7.6 | 6.8 | 5.7 | -1.18 | 55 | 56 | 58 | 59 | 378 | 116 |
| Breton^x^ | D | 26498 | 7.5 | 5.1 | 1.3 | 1.2 | -0.10 | 205 | 167 | 182 | 131 | 4026 | 671 |
| Camarguais^x^ | W | 5244 | 10.4 | 2.6 | 0.9 | 3.3 | 2.38 | 144 | 128 | 22 | 46 | 969 | 187 |
| Cob normand^x^ | D | 4058 | 8.8 | 3.9 | 1.6 | 0.9 | -0.75 | 120 | 88 | 173 | 97 | 398 | 122 |
| Comtois^x^ | D | 14233 | 6.8 | 1.9 | 0.2 | 0.2 | 0.04 | 520 | 395 | 189 | 257 | 3779 | 520 |
| Highland | P | 393 | 10.8 | 5.5 | 3.7 | 2.3 | -1.41 | 73 | 44 | 100 | 118 | 111 | 60 |
| Landais^x^ | P | 827 | 13.7 | 3.1 | 4.6 | 3.7 | -1.01 | 33 | 44 | 29 | 33 | 159 | 53 |
| Merens^x^ | W | 6399 | 10.0 | 5.7 | 5.6 | 5.4 | -0.18 | 51 | 799 | 45 |  | 598 | 105 |
| Percheron^x^ | D | 9830 | 7.8 | 4.4 | 0.9 | 0.6 | -0.24 | 258 | 160 | 275 | 191 | 1188 | 377 |
| Poitevin^x^ | D | 684 | 7.9 | 6.7 | 7.5 | 5.6 | -1.98 | 43 | 53 | 50 | 156 | 210 | 77 |
| Pottock^x^ | P | 1363 | 8.8 | 2.2 | 0.8 | 0.9 | 0.10 | 145 | 76 | 77 | 67 | 688 | 361 |
| Selle français | W | 65753 | 11.6 | 2.9 | 0.9 | 0.5 | -0.37 | 172 | 83 | 213 | 134 | 4757 | 397 |
| Shetland | P | 4992 | 9.3 | 4.9 | 0.5 | 1.7 | 1.23 | 511 | 132 | 113 | 132 | 2940 | 1686 |
| Trait du Nord^x^ | D | 833 | 7.5 | 3.5 | 1.6 | 0.5 | -1.12 | 109 | 70 | 321 | 189 | 159 | 109 |
| Trotteur français | W | 125913 | 11.2 | 6.2 | 2.4 | 2.0 | -0.33 | 131 | 541 | 129 | 181 | 5077 | 565 |
| Welsh Pony | P | 3665 | 11.1 | 5.1 | 1.0 | 3.3 | 2.39 | 276 | 131 | 63 | 51 | 3264 | 2022 |

*Pref,* reference population size; *T*, average generation length in years; *EqG*, number of equivalent generations; *F_IS_*, fixation index; *N_eCi_*, *N_e_* based on individual coancestry rate model; *N_eCt_*, *N_e_* based on coancestry rate between two successive generations model; *N_eFi_*, *N_e_* based on individual inbreeding rate model; *N_eFt_*, *N_e_* based on inbreeding rate between two successive generations model; *N_es_*, *N_e_* based on sex ratio based model; *N_ev_*, *N_e_* based on variance of progeny size model; W, warm-blooded breed; D, draught-horse breed; P, pony breed; ^x^ breeds receiving endangered breed subsidies.

Table S4: Genealogical parameters and effective population sizes for the 60 dog breeds

| Breed | Type | *Pref* | *T* | *EqG* | *C* (%) | *F* (%) | *IBD* | | | | *N_es_* | *N_ev_* |
| --- | --- | --- | --- | --- | --- | --- | --- | --- | --- | --- | --- | --- |
|  |  |  |  |  |  |  | *N_eCi_* | *N_eCt_* | *N_eFi_* | *N_eFt_* |  |  |
| American Staffordshire Terrier | 16166 | 3.0 | 5.8 | 2.2 | 4.6 | 2.43 | 135 |  | 50 | 84 | 2752 | 471 |
| Ariégeois | 2449 | 4.3 | 4.3 | 1.8 | 3.4 | 1.62 | 115 | 123 | 49 | 50 | 621 | 238 |
| Australian Shepherd | 4696 | 3.3 | 4.1 | 1.2 | 1.2 | 0.01 | 174 | 302 | 149 | 115 | 658 | 157 |
| Barbet | 110 | 4.7 | 4.0 | 9.1 | 7.9 | -1.29 | 21 | 17 | 22 |  | 37 | 35 |
| Basset fauve de Bretagne | 4011 | 4.0 | 7.2 | 2.4 | 4.2 | 1.83 | 153 | 232 | 75 | 157 | 1136 | 554 |
| Beagle | 10258 | 4.3 | 8.0 | 2.0 | 4.6 | 2.69 | 203 | 335 | 75 | 83 | 2507 | 830 |
| Bearded Collie | 2612 | 4.9 | 5.1 | 2.3 | 4.5 | 2.32 | 113 | 80 | 48 | 28 | 343 | 221 |
| Beauceron | 16549 | 4.4 | 9.2 | 3.7 | 6.2 | 2.55 | 121 | 90 | 64 | 54 | 2160 | 526 |
| Belgian Shepherd Dog Malinois | 18529 | 5.1 | 6.8 | 2.1 | 4.3 | 2.24 | 162 | 153 | 66 | 68 | 2913 | 663 |
| Berger des Pyrénées | 3678 | 4.9 | 6.9 | 6.0 | 8.8 | 3.00 | 57 | 82 | 33 | 34 | 637 | 213 |
| Bernese Mountain Dog | 9114 | 3.6 | 5.2 | 1.3 | 1.9 | 0.64 | 209 | 222 | 115 | 89 | 1263 | 346 |
| Border Collie | 5384 | 4.3 | 3.2 | 0.8 | 0.9 | 0.05 | 190 | 184 | 143 | 106 | 1120 | 354 |
| Borzoi | 1137 | 4.3 | 5.2 | 1.2 | 3.1 | 1.88 | 213 | 109 | 74 | 47 | 345 | 267 |
| Bouledogue français | 10106 | 3.0 | 7.8 | 2.4 | 3.2 | 0.83 | 165 | 372 | 108 | 180 | 2048 | 389 |
| Braque Saint-Germain | 349 | 5.0 | 8.0 | 8.8 | 7.5 | -1.37 | 43 | 34 | 44 | 16 | 82 | 59 |
| Bull Terrier | 2374 | 3.1 | 4.3 | 1.4 | 1.1 | -0.25 | 160 | 84 | 145 | 111 | 524 | 241 |
| Bulldog | 2811 | 3.1 | 4.3 | 0.8 | 1.4 | 0.61 | 276 | 167 | 127 | 105 | 798 | 330 |
| Cairn Terrier | 6450 | 4.1 | 6.8 | 1.7 | 3.1 | 1.37 | 196 | 189 | 92 | 145 | 912 | 391 |
| Cavalier King Charles Spaniel | 20803 | 3.6 | 6.8 | 1.4 | 3.3 | 1.96 | 246 | 388 | 84 | 93 | 3322 | 612 |
| Chien de Montagne des Pyrénées | 1681 | 4.4 | 6.7 | 3.7 | 6.3 | 2.69 | 90 | 37 | 41 | 18 | 277 | 211 |
| Collie Rough | 5001 | 3.9 | 5.8 | 1.3 | 4.1 | 2.82 | 219 | 177 | 60 | 62 | 914 | 480 |
| Coton de Tuléar | 7395 | 4.1 | 6.3 | 4.1 | 6.4 | 2.40 | 77 | 76 | 40 | 48 | 851 | 303 |
| Czeslovakian Wolfdog | 178 | 3.1 | 3.1 | 3.1 | 0.4 | -2.87 | 48 | 19 | 392 | 142 | 65 | 80 |
| Dalmatian | 3508 | 4.1 | 5.7 | 2.0 | 2.3 | 0.33 | 143 | 98 | 88 | 53 | 526 | 221 |
| Dobermann | 8007 | 4.0 | 4.6 | 1.0 | 2.3 | 1.32 | 222 | 240 | 79 | 60 | 1172 | 393 |
| Dogo Argentino | 4335 | 3.0 | 4.2 | 1.0 | 1.6 | 0.58 | 210 | 251 | 110 | 54 | 778 | 284 |
| Dogue de Bordeaux | 2787 | 3.2 | 7.2 | 3.4 | 4.0 | 0.64 | 109 | 70 | 83 | 49 | 538 | 163 |
| English Cocker Spaniel | 19010 | 4.0 | 6.7 | 0.6 | 2.3 | 1.69 | 536 | 533 | 127 | 91 | 2519 | 912 |
| English Pointer | 7566 | 4.8 | 5.8 | 1.6 | 2.4 | 0.85 | 176 | 149 | 97 | 52 | 1693 | 538 |
| English Setter | 26018 | 4.9 | 6.5 | 1.3 | 2.2 | 0.86 | 245 | 478 | 122 | 141 | 4800 | 713 |
| English Springer Spaniel | 7454 | 4.3 | 6.4 | 1.8 | 2.9 | 1.17 | 179 | 553 | 93 | 510 | 1317 | 456 |
| Epagneul Breton | 25477 | 4.6 | 9.2 | 3.3 | 5.2 | 1.99 | 138 | 175 | 75 | 74 | 3943 | 839 |
| German Boxer | 9245 | 3.6 | 5.4 | 1.3 | 2.5 | 1.21 | 214 | 294 | 92 | 84 | 1813 | 489 |
| German Shepherd Dog | 46752 | 4.1 | 5.2 | 0.6 | 1.9 | 1.23 | 404 | 749 | 120 | 83 | 6041 | 1212 |
| German Short-haired Pointing Dog | 8219 | 4.9 | 7.5 | 2.5 | 3.5 | 1.05 | 153 | 121 | 93 | 76 | 1381 | 553 |
| German Spitz | 2529 | 4.4 | 7.0 | 2.3 | 6.8 | 4.70 | 168 | 53 | 43 | 24 | 859 | 412 |
| Golden Retriever | 28383 | 3.8 | 4.9 | 0.7 | 1.4 | 0.70 | 374 | 548 | 146 | 73 | 2714 | 518 |
| Great Dane | 5764 | 3.4 | 7.4 | 1.2 | 4.6 | 3.40 | 310 | 203 | 72 | 72 | 1256 | 690 |
| Griffon bleu de Gascogne | 3103 | 3.7 | 4.0 | 1.7 | 2.3 | 0.60 | 121 | 110 | 77 | 63 | 761 | 333 |
| Griffon d'arrêt à poil dur Korthals | 6769 | 5.1 | 8.5 | 4.3 | 5.6 | 1.28 | 96 | 78 | 65 | 57 | 950 | 245 |
| Irish Red Setter | 3129 | 5.0 | 6.3 | 2.1 | 5.9 | 3.87 | 153 | 120 | 47 | 30 | 513 | 249 |
| Italian Corso Dog | 5368 | 2.7 | 3.8 | 0.9 | 1.5 | 0.54 | 201 | 400 | 93 | 60 | 832 | 129 |
| King Charles Spaniel | 881 | 3.4 | 6.0 | 3.0 | 2.7 | -0.25 | 98 | 151 | 92 | 66 | 272 | 121 |
| Labrador Retriever | 33146 | 4.1 | 5.5 | 0.7 | 2.3 | 1.63 | 419 | 677 | 97 | 64 | 3989 | 1176 |
| Leonberger | 4822 | 4.2 | 6.7 | 2.4 | 2.8 | 0.49 | 145 | 96 | 110 | 111 | 799 | 358 |
| Newfoundland | 5998 | 3.9 | 4.9 | 1.1 | 2.6 | 1.49 | 232 | 195 | 80 | 47 | 893 | 493 |
| Parson/Jack Russell Terrier | 10625 | 3.5 | 3.4 | 1.3 | 2.2 | 0.95 | 139 | 250 | 63 | 59 | 1718 | 241 |
| Poodle | 8104 | 4.6 | 6.2 | 0.5 | 4.9 | 4.43 | 692 | 457 | 51 | 34 | 1714 | 1138 |
| Romagna Water Dog | 178 | 2.8 | 3.0 | 2.9 | 0.4 | -2.57 | 50 | 24 | 231 | 130 | 78 | 51 |
| Rottweiler | 20161 | 3.7 | 4.8 | 0.6 | 1.7 | 1.08 | 415 | 1451 | 111 | 99 | 3360 | 799 |
| Saarloos Wolfdog | 201 | 5.0 | 3.1 | 4.3 | 3.9 | -0.38 | 36 | 18 | 29 | 15 | 63 | 67 |
| Shar Pei | 3970 | 3.4 | 4.9 | 1.2 | 3.0 | 1.76 | 202 | 159 | 71 | 45 | 790 | 380 |
| Shih Tzu | 8846 | 4.0 | 6.0 | 0.8 | 2.8 | 1.98 | 386 | 684 | 92 | 160 | 1707 | 870 |
| Short-haired Weimaraner | 6083 | 4.5 | 8.5 | 4.8 | 5.7 | 1.01 | 87 | 68 | 64 | 42 | 997 | 332 |
| Siberian Husky | 4060 | 4.8 | 5.0 | 0.9 | 2.7 | 1.88 | 278 | 138 | 76 | 42 | 909 | 700 |
| Smooth-haired Dachshund | 2890 | 4.7 | 5.9 | 1.2 | 5.1 | 3.97 | 250 | 128 | 48 | 48 | 664 | 433 |
| West Highland White Terrier | 10736 | 3.9 | 6.0 | 0.9 | 2.3 | 1.43 | 339 | 338 | 109 | 72 | 1708 | 673 |
| Whippet | 4385 | 4.9 | 5.6 | 1.2 | 3.6 | 2.39 | 231 | 112 | 68 | 61 | 1049 | 709 |
| Wire-haired Dachshund | 10647 | 3.8 | 6.1 | 0.8 | 3.3 | 2.48 | 364 | 300 | 75 | 38 | 2877 | 948 |
| Yorkshire Terrier | 20547 | 4.1 | 6.7 | 0.9 | 3.4 | 2.49 | 370 | 295 | 82 | 78 | 4042 | 1443 |

*Pref,* reference population size; *T*, average generation length in years; *EqG*, number of equivalent generations; *F_IS_*, fixation index; *N_eCi_*, *N_e_* based on individual coancestry rate model; *N_eCt_*, *N_e_* based on coancestry rate between two successive generations model; *N_eFi_*, *N_e_* based on individual inbreeding rate model; *N_eFt_*, *N_e_* based on inbreeding rate between two successive generations model; *N_es_*, *N_e_* based on sex ratio based model; *N_ev_*, *N_e_* based on variance of progeny size model

Table S5: Residual standard deviations according to methods and species

| Species | *IBD* methods | | | | *Ne_s_* | *Ne_v_* |
| --- | --- | --- | --- | --- | --- | --- |
|  | *Ne_Ci_* | *Ne_Ct_* | *Ne_Fi_* | *Ne****_Ft_*** |  |  |
| Cattle | 253 | 71 | 145 | 59 | 36268 | 1110 |
| Sheep | 116 | 29 | 136 | 74 | 2471 | 398 |
| Horse | 143 | 193 | 86 | 61 | 1969 | 562 |
| Dog | 126 | 239 | 54 | 68 | 1277 | 313 |
