# Supplementary material for: Methods to estimate effective population size using pedigree data: Examples in dog, sheep, cattle and horse
Source: Genet Sel Evol. 2013 Jan 2;45(1):1. doi: 10.1186/1297-9686-45-1 (PMC3599586; doi:10.1186/1297-9686-45-1)
Supplement: Additional file 3 — Relation between equivalent complete generations traced EqG and inbreeding F (Figure S1), EqG and coancestry C (Figure S2), F and C (Figure S3) for the 140 breeds. Figures S1, S2 and S3 show relations between genealogical indicators according to species studied. [file 1297-9686-45-1-S3.doc]

Coancestry *C*

Inbreeding *F*

Coancestry *C*

Equivalent complete generations traced (*EqG*)

Equivalent complete generations traced (*EqG*)

Inbreeding *F*

Figure S1

Figure S2

Figure S3

♦ cattle breeds

○ sheep breeds

▲ horse breeds

X dog breeds
