# Supplementary material for: Methods to estimate effective population size using pedigree data: Examples in dog, sheep, cattle and horse
Source: Genet Sel Evol. 2013 Jan 2;45(1):1. doi: 10.1186/1297-9686-45-1 (PMC3599586; doi:10.1186/1297-9686-45-1)
Supplement: Additional file 4 — Correlation circles from the principal component analysis considering the six effective population sizes computed for the four species independently. This figure shows correlations circle between computation methods and the two first components, after PCA have been independently performed for each of the four species. [file 1297-9686-45-1-S4.doc]

Axis 2 (27%)

Axis 2 (23%)

Axis 2 (15%)

Axis 2 (29%)

Axis 1 (72%)

Axis 1 (53%)

Axis 1 (48%)

Axis 1 (51%)

Figure S3: **Horse**

Figure S4: **Dog**

*N_eCi_*

*N_eCt_*

*N_eFi_*

*N_eFt_*

*N_es_*

*N_ev_*

*N_eCi_*

*N_eCt_*

*N_eFi_*

*N_eFt_*

*N_es_*

*N_ev_*

*N_eCi_*

*N_eCt_*

*N_eFi_*

*N_eFt_*

*N_es_*

*N_ev_*

*N_eCi_*

*N_eCt_*

*N_eFi_*

*N_eFt_*

*N_es_*

*N_ev_*

Figure S2: **Sheep**

Figure S1: **Cattle**
